# Individual Responses to Completion of Short-Term and Chronic Interval Training: A Retrospective Study

**DOI:** 10.1371/journal.pone.0097638

**Published:** 2014-05-21

**Authors:** Todd A. Astorino, Matthew M. Schubert

**Affiliations:** 1 Department of Kinesiology, California State University San Marcos, San Marcos, California, United States of America; 2 School of Allied Health Sciences, Griffith University, Gold Coast, Queensland, Australia; Purdue University, United States of America

## Abstract

Alterations in maximal oxygen uptake (VO_2_max), heart rate (HR), and fat oxidation occur in response to chronic endurance training. However, many studies report frequent incidence of “non-responders” who do not adapt to continuous moderate exercise. Whether this is the case in response to high intensity interval training (HIT), which elicits similar adaptations as endurance training, is unknown. The aim of this retrospective study was to examine individual responses to two paradigms of interval training. In the first study (study 1), twenty active men and women (age and baseline VO_2_max = 24.0±4.6 yr and 42.8±4.8 mL/kg/min) performed 6 d of sprint interval training (SIT) consisting of 4–6 Wingate tests per day, while in a separate study (study 2), 20 sedentary women (age and baseline VO_2_max = 23.7±6.2 yr and 30.0±4.9 mL/kg/min) performed 12 wk of high-volume HIT at workloads ranging from 60–90% maximal workload. Individual changes in VO_2_max, HR, and fat oxidation were examined in each study, and multiple regression analysis was used to identify predictors of training adaptations to SIT and HIT. Data showed high frequency of increased VO_2_max (95%) and attenuated exercise HR (85%) in response to HIT, and low frequency of response for VO_2_max (65%) and exercise HR (55%) via SIT. Frequency of improved fat oxidation was similar (60–65%) across regimens. Only one participant across both interventions showed non-response for all variables. Baseline values of VO_2_max, exercise HR, respiratory exchange ratio, and body fat were significant predictors of adaptations to interval training. Frequency of positive responses to interval training seems to be greater in response to prolonged, higher volume interval training compared to similar durations of endurance training.

## Introduction

Results from recent randomized controlled studies indicate that individual variability exists in magnitude of response to prolonged endurance training. Data from young [1] and older men and women [2] demonstrate marked variability in magnitude of change in maximal oxygen uptake (VO_2_max) to endurance training. Data from the HERITAGE study [3] revealed a mean increase in VO_2_max equal to 400 mL/min, yet individual responses ranged from minimal improvement to as great as 1.0 L/min. In the DREW study [4], 44.9, 23.8, and 19.3% of postmenopausal women showed no change in VO_2_max in response to 6 mo of one of three aerobic exercise regimens (energy expenditure equal to 4, 8, and 12 kcal/kg/wk). More recently, Scharhag-Rosenberger et al. [5] documented similar individual variability in change in VO_2_max (−0.38–0.87 L/min) and exercise HR (−22.0–2.0 b/min) in untrained individuals completing 1 yr of endurance training at 60% heart rate reserve, with 24% and 17% of participants showing no training-induced changes in these parameters. In this study, only 55% of participants (10/18) displayed meaningful increases in both parameters with training. An explanation for these results is related to genetics, as it has been reported that 47% and 34% of the change in VO_2_max [3] and exercise HR [6] is heritable. In addition, baseline values of VO_2_max have been shown to be associated with training-inducedchanges in some studies [4] but not others [2], [7]. Clearly, there is marked heterogeneity in adaptation to chronic endurance training, which highlights the need to tailor exercise prescription to every individual to promote adaptation.

One parameter not examined in these studies is whole-body fat oxidation, which not only contributes to energy metabolism but the dysfunction of which is related to risk of obesity, insulin resistance, and diabetes [8], [9]. Fat oxidation is typically increased in response to endurance training due to increased activities of carnitine acyl transferase I (CAT-1), lipase, and/or hydroxyl acyl dehydrogenase (β-HAD) [10], enhanced mitochondrial mass [11], and greater muscle fatty acid binding protein content [12]. In trained athletes, Goedecke et al. [13] demonstrated that fat oxidation during exercise, as represented by respiratory exchange ratio (RER), was determined by variables including muscle glycogen content, ratio of type I fibers, training volume, and blood lactate and free fatty acid concentration. In addition, fat oxidation is enhanced in response to low-volume sprint interval training (SIT) [14] as well as short-term [15] and more prolonged regimens [16] of high intensity interval training (HIT) characterized by completion of brief, repeated bouts of intense exercise separated by recovery.

Interval training has become a widely-employed modality of exercise training in populations including young, active men and women [17] and individuals with obesity [18], diabetes [19], and heart disease [20]. However, little is known about the magnitude of individual responses to interval training, which over the last decade has been shown to elicit similar [21], [22], and in some cases, superior adaptations [20], [23] versus continuous endurance training while being extremely time-efficient. Moreover, interval training has been reported [24] to be more enjoyable than continuous exercise despite exercising at higher intensities, which in the long run may promote greater adherence to training and on an individual level, superior maintenance of fitness level, health status, and quality of life. Despite over 100 studies being published in the last decade concerning effects of interval training on variables including VO_2_max and body composition, no study has attempted to elucidate individual responses to interval training. Therefore, the aim of the present study was to identify individual “responders” and “non-responders” for to variables related to metabolic health (VO_2_max and lipid oxidation) and cardiovascular function (exercise HR) in response to two commonly used modalities of interval training previously-employed in our lab [14], [16], [25]. It was hypothesized that frequency of “non-responders” would be less than that typically reported after endurance training [4], [5]. Ultimately, the development of individualized exercise prescription using this novel approach may help optimize responses to training and overall health status of various individuals.

## Method

### Ethics Statement

Prior to providing written informed consent, all participants filled out a health-history questionnaire to ensure that they met all inclusion criteria, and all procedures were approved by the CSU–San Marcos University Institutional Review Board.

### Participants

Twenty habitually-active men and women participated in study 1, which examined potential gender differences in adaptation to short-term low-volume interval training. Mean age, body fat, current physical activity, and VO_2_max were equal to 24.0±4.6 yr, 20.3±4.7%, 7.9±2.0 h/wk, and 42.8±4.8 mL/kg/min, respectively. Study 2 was designed to examine the timecourse of changes in metabolic health in response to two doses of prolonged interval training in 20 non-obese sedentary women free of disease. They initially completed a validated questionnaire (Past Year Total Physical Activity Questionnaire) to confirm that they completed ≤1 h/wk of formal physical activity in the preceding year. Their age, body fat, and VO_2_max were equal to 23.7±6.2 yr, 24.2±5.8%, and 30.0±4.9 mL/kg/min, respectively.

### Design

In study 1, recreationally-active men and women underwent 2 wk of Wingate-based SIT [14], [18], [21]. At baseline and after completion of training, measures of VO_2_max, HR, and lipid oxidation were determined on separate days at least 24 h apart. Participants were required to maintain their habitual training status which was confirmed with a training log, and time of day was standardized within subjects across all trials. In study 2, sedentary young women completed 12 wk of a more tolerable form of interval training [15], [19] at intensities equal to 60–80% or 80–90%Wmax, during which these variables were assessed at baseline and every 3 wk of the study over two separate sessions. They were required to refrain from additional physical activity other than activities of daily living outside of the study. Exercise was performed at approximately the same time of day (≤60 min) within participants. In both studies, body composition was assessed pre- and post-training using waist:hip ratio and sum of three skinfolds (chest, abdomen, thigh for men and triceps, suprailiac, and thigh for women) following standardized procedures [26].

### Interval Training

Following procedures described in previous studies [14], [18], [21], men and women in study 1 performed six sessions of low volume SIT consisting of repeated Wingate tests (4–6 per day at intensities = 200–300%Wmax) over a 2 wk period. Resistance was equal to 7.5%BW for women and 8.5%BW for men. Training was performed on the Monark peak bike (model 894e, Vansbro, Sweden) and consisted of 30 s of “all-out” cycling interspersed with 5 min of unloaded pedaling. Results [14] showed that peak power, mean power, and minimum power were increased (p<0.05) with SIT, although no change in fatigue index ([peak power – minimum power/peak power]×100) was demonstrated. In study 2, women performed 3 d/wk of interval training for 12 wk consisting of six to ten 1 min bouts of cycling at work rates equal to 60–80%Wmax or 80–90%Wmax, similar to previously performed [19]. All training was performed on an electrically-braked cycle ergometer (Velotron Dynafit Pro, RacerMate, Seattle, WA). Each week, number of bouts (+2 bouts per week) and work rate (+5%Wmax) were increased to promote progression [25]. By the end of training, women were training at absolute work rates 25% higher than that completed at baseline [25].

### Assessment of VO_2_max, HR, and Fat Oxidation

In study 1, VO_2_max was assessed on a cycle ergometer (Monark 828e, Vansbro, Sweden) during which pulmonary gas exchange data were continuously obtained using a metabolic cart (ParvoMedics True One, Sandy, UT). The system was calibrated to gases of known concentration as well as to room air, and a 3-L syringe (Hans Rudolph, Kansas City, MO) was used to calibrate volume. Work rate began at 70 W for the initial 2 min, followed by 28 W/min increases in power output until volitional exhaustion. Test duration ranged from 8–12 min [27], and attainment of a plateau in VO_2_, HRmax±10 b/min of 220– age, and RERmax >1.15 were used to verify VO_2_max attainment [28], [29]. The coefficient of variation for VO_2_max derived from controls in this study as well as other active men and women completing repeated bouts of graded exercise testing following these methods was <2.8%, comparable to other studies [29]. At least 24 h later at the same time of day and 3 h post-absorptive, participants completed 10 min of cycling at each of three intensities equal to 50, 60, and 70%Wmax, during which gas exchange data were continuously obtained. Nutritional intake was standardized for 24 h before this bout and assessed via diet records. Data were averaged every 5 min and used to calculate RER and rates of fat and carbohydrate oxidation (in kcal/min) using the Frayn equations [30]. Coefficient of variation for exercise RER at these work rates obtained from active men and women was equal to 4.3%. Intraclass correlations for RER at 50, 60, and 70%Wmax were equal to 0.63, 0.82, and 0.87, respectively, similar to a previous study in trained cyclists [13]. Heart rate was obtained continuously through telemetry (Polar Electro, Woodbury, NY) and averaged every 5 min during exercise. Coefficient of variation in exercise HR was equal to 2.1%.

In study 2, VO_2_max was assessed on a cycle ergometer (Velotron DynaFit Pro, RacerMate, Seattle, WA) with simultaneous measurement of gas exchange data (ParvoMedics True One, Sandy, UT). Power output was equal to 40 W for the initial 2 min and increased in a ramp-like manner by 20 W/min until volitional exhaustion. Coefficient of variation for sedentary women completing ramp cycle ergometry in our lab is <3.0%. Similar criteria were used to confirm attainment of VO_2_max [28]. Women returned at least 24 h later at the same time of day after an overnight fast and completed graded cycling (40 W for 4 min followed by 20 W/min increases in work rate every 3 min) until RER exceeded 1.0 for an entire stage. Nutritional intake was standardized for 24 h before this bout. Gas exchange data and HR were averaged from the last 2 min of each stage, with the former used to determine RER and fat and carbohydrate oxidation using the Frayn equations [30]. Coefficients of variation for these measures were equal to 4.6% for RER and 3.4% for exercise HR, respectively.

### Assessing Individual Responses

In study 1, change in VO_2_max (expressed as a percent as well as in L/min and mL/kg/min) was computed in response to 2 wk of Wingate-based SIT, as in some participants, body mass did change during the study. Changes in HR and lipid oxidation derived from RER were obtained from a continuous bout of cycling for 10 min at 50, 60, and 70%Wmax. Total changes in HR (b/min) and lipid oxidation (RER) were added across these three workloads and then divided by 3 to identify an average change in these variables in response to training. For example, if HR and RER were reduced by −3, −6, and −4 b/min and −0.02, −0.04, and −0.06 at 50, 60, and 70%Wmax in response to training, change in HR and RER was equal to −4.3 b/min and −0.04 (13% greater fat oxidation), respectively. In study 2, change in VO_2_max was determined by comparing determinations of VO_2_max before (0 wk) and after training (12 wk), and expressed as a percent change as well as in L/min and mL/kg/min, respectively. Training-induced changes in HR and lipid oxidation were obtained using similar procedures as performed in study 1. All women performed at least 4 stages of progressive cycling exercise at work rates equal to 40, 60, 80, and 100 W.

### Data Analysis

Results are reported as mean ± SD and were analyzed using SPSS Version 20.0 (Chicago, IL). Confidence intervals (95%) were also computed for select variables. Multiple regression was used to determine predictors of the change in VO_2_max (%), exercise HR (b/min), and fat oxidation (% change in fat oxidation according to RER) in response to SIT and HIT. Based on previous findings [4]–[5], [7], [13], variables entered in each two-predictor model included baseline values of VO_2_max (mL/kg/min), exercise HR (b/min) and fat oxidation (% fat oxidation from mean RER value), age, %BF, and related parameters obtained in both studies. Responders were identified by a magnitude of adaptation greater than 1 CV for that parameter, and participants with changes from baseline less than 1 CV were labeled as “nonresponders” as previously-reported [5]. Statistical significance was established as p<0.05.

## Results

### Study 1

#### Individual changes in VO_2_max

**Figure 1:**
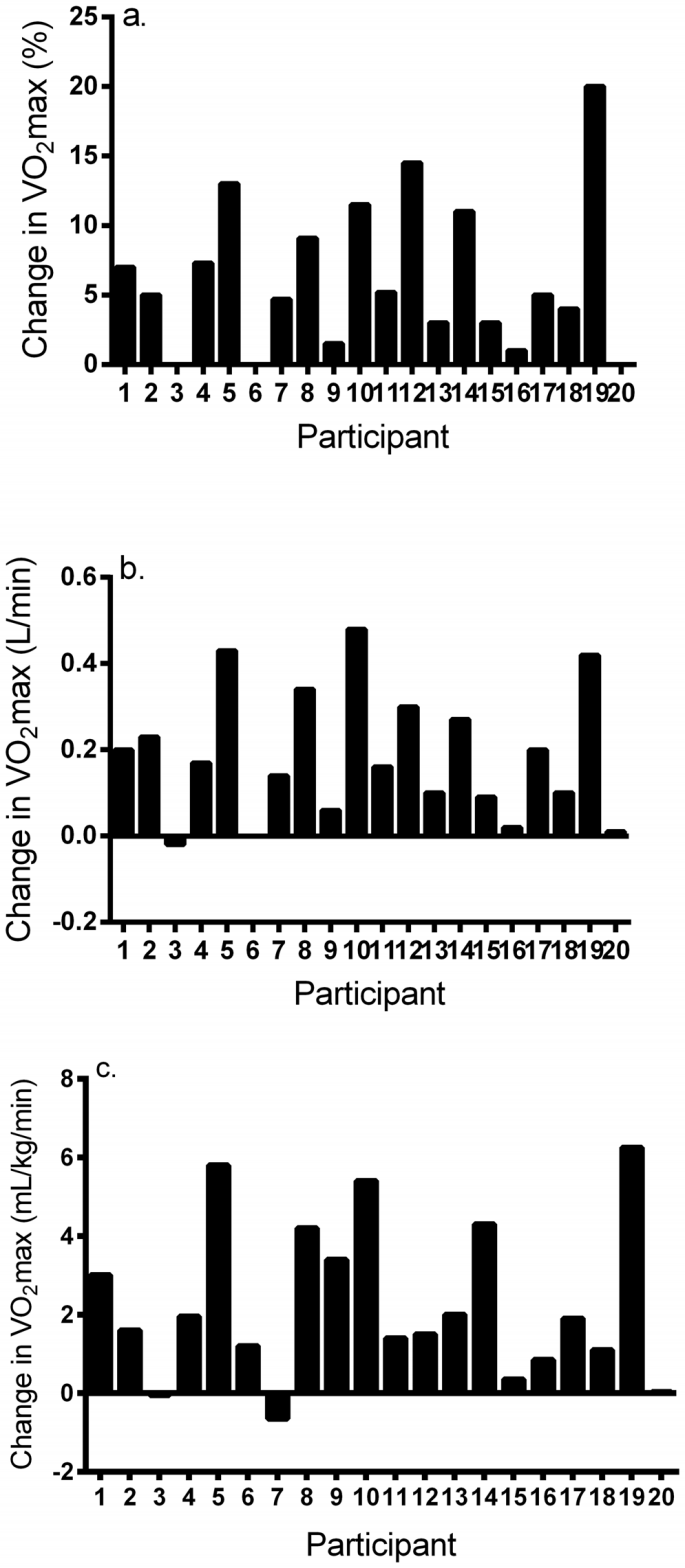
Individual responses for a) percent change in VO_2_max; b) absolute change in VO_2_max; and c) relative change in VO_2_max in response to 2 wk of low-volume interval training.

There was 100% compliance to training in this study. Figure 1a–c shows change in VO_2_max across subjects. The mean (± SD) and 95% confidence interval for percent change, absolute, and relative increase in VO_2_max was equal to 6.3±5.4% (3.7–8.8%, range = 0–20%), 0.19±0.13 L/min (0.12–0.26 L/min, range = −0.02 = 0.48 L/min), and 2.6±2.0 mL/kg/min (1.3–3.2 mL/kg/min, range = −0.65–6.25 mL/kg/min), respectively. Overall, 13 of 20 participants (65%) showed meaningful improvements (>2.8%) in VO_2_max in response to 2 wk of SIT, with four individuals (20%) showing no change and three (15%) showing insignificant increases in VO_2_max.

#### Individual changes in exercise HR

**Figure 2:**
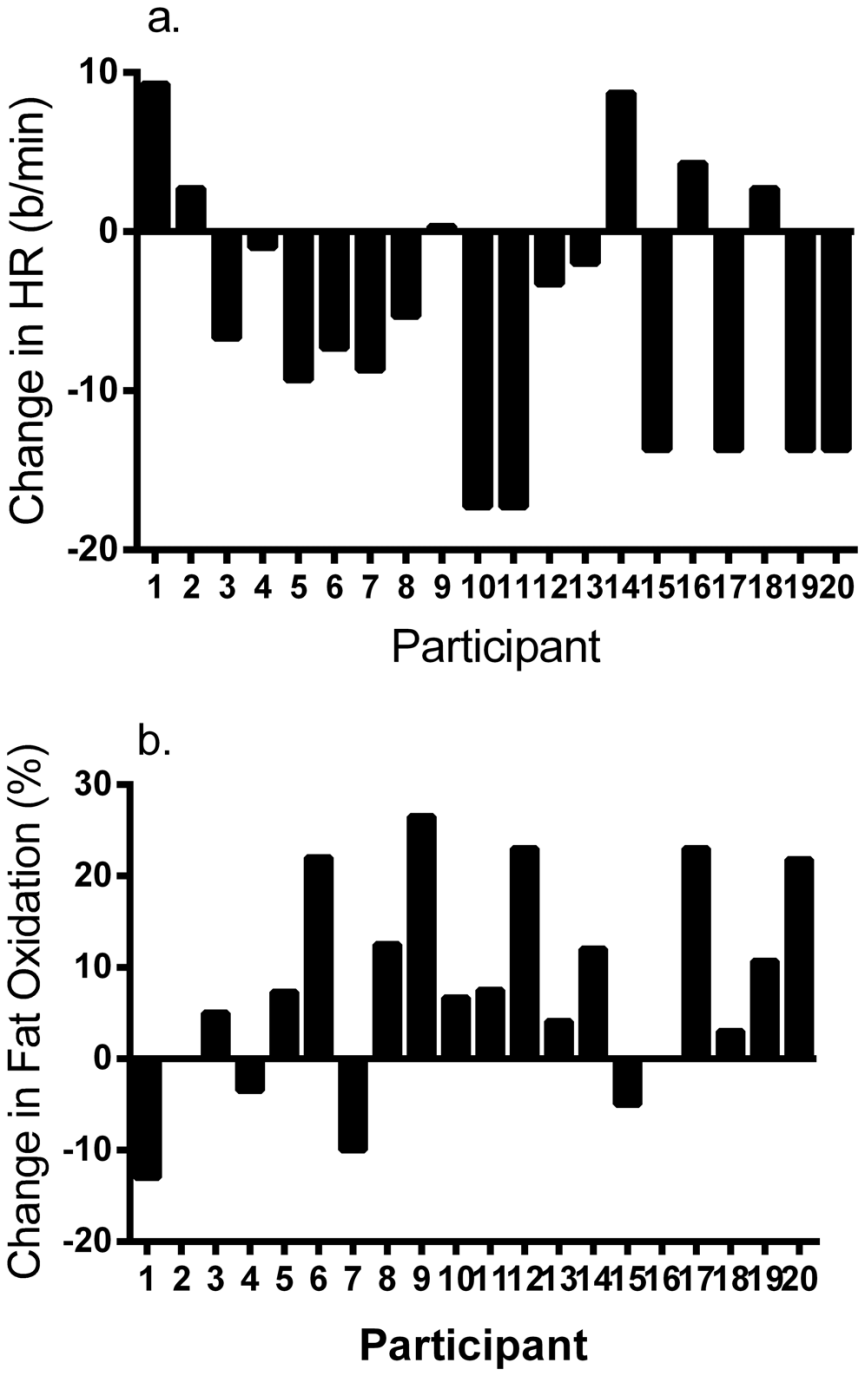
Individual responses for a) change in exercise HR and b) lipid oxidation in response to 2 wk of low-volume interval training.

Mean absolute and percent change in exercise HR was equal to −5±8 b/min (95%CI = −9–−1 b/min, range = −17–9 b/min) and −2.8±4.5% (−4.9 = −0.7%, range = −9.1–5.3%), respectively. Eleven of 20 participants (55%) showed reductions in exercise HR, with 25% showing higher HR and the remaining 20% presenting insignificant changes in HR in response to training. These data are revealed in Figure 2a.

#### Individual changes in fat oxidation

Mean change in fat oxidation was equal to 7.7±11.4% (2.3–13.0%, range = −13.0–26.5%). Sixty percent of men and women revealed improvements in fat oxidation; whereas, 25% showed no change and 15% demonstrated reduced fat oxidation in response to SIT (Figure 2b).

#### Multiple regression data

Two-predictor models were used to identify predictors of the change in each parameter in response to Wingate-based SIT. A model (R = 0.61, p = 0.03) consisting of baseline VO_2_max (r = −0.44, p = 0.03) and Wingate-derived fatigue index (r = 0.50, p = 0.01) explained 36% of the percent change in VO_2_max in response to training,. Age (r = 0.67, p = 0.001) and baseline HR at 30 min of cycling explained the greatest variance in change in exercise HR (R = 0.68, R^2^ = 0.46, p<0.01), with age serving as an independent predictor of exercise HR (t = 3.84, p = 0.001). A model (R = 0.52, p = 0.03) including age (r = −0.41, p = 0.03) and current physical activity (r = −0.47, p = 0.02) explained 27% of the change (R = 0.52, p = 0.03) in fat oxidation.

### Study 2

Despite somewhat different intensities of HIT performed in this study, no training-induced differences in any parameter were observed between regimens, so data were combined. Compliance to training was high (96.4% of all required sessions).

#### Individual changes in VO_2_max

**Figure 3:**
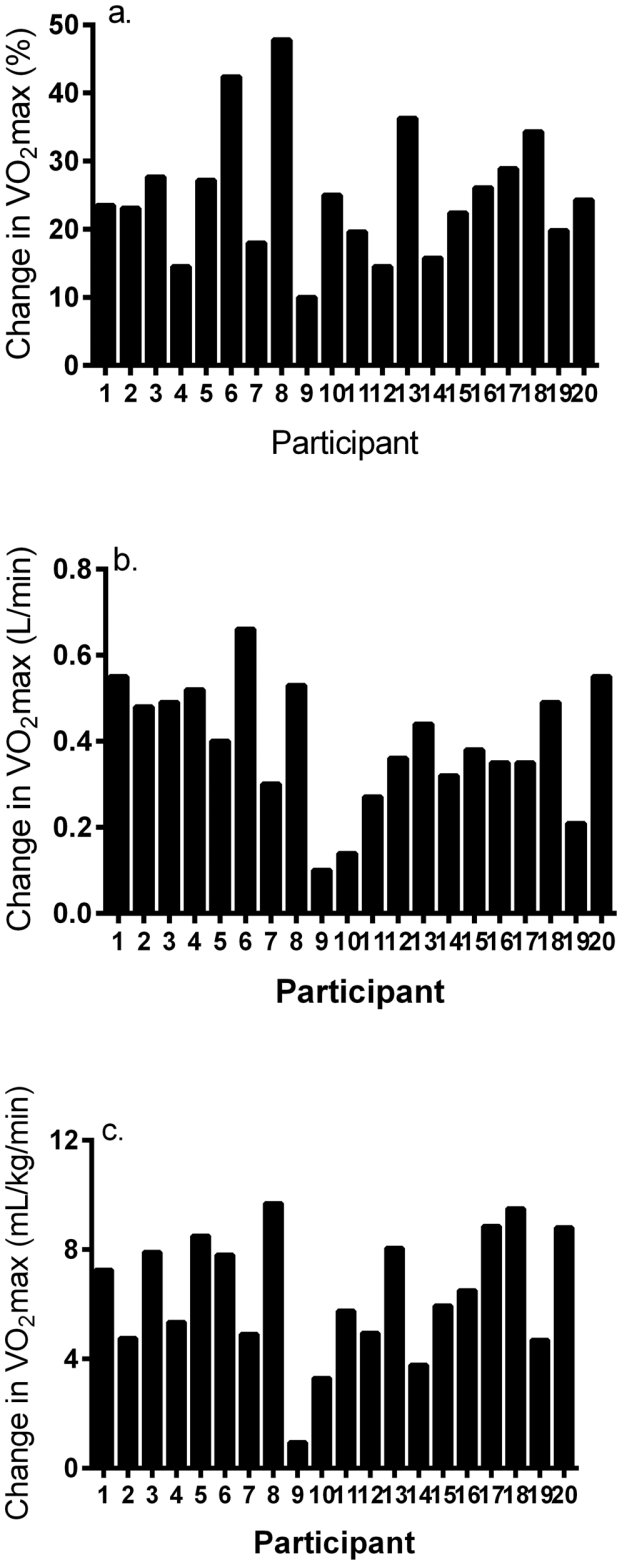
Individual responses for a) percent change in VO_2_max; b) absolute change in VO_2_max; and c) relative change in VO_2_max in response to 12 wk of high-volume interval training.

Mean change in VO_2_max was equal to 25.1±9.5% (95%CI = 20.6–29.5%, range = 2.7–47.8%). Absolute and relative change in VO_2_max was equal to 0.39±0.16 L/min (95%CI = 0.32–0.46 L/min, range = 0.08−0.66 L/min) and 6.4±2.3 mL/kg/min (5.3–7.4 mL/kg/min, range = 0.9−9.7 mL/kg/min), respectively. With exception of one woman, all remaining (95%) participants were classified as responders showing an increase in VO_2_max via HIT. These data are revealed in Figure 3a–c.

#### Individual changes in exercise HR

**Figure 4:**
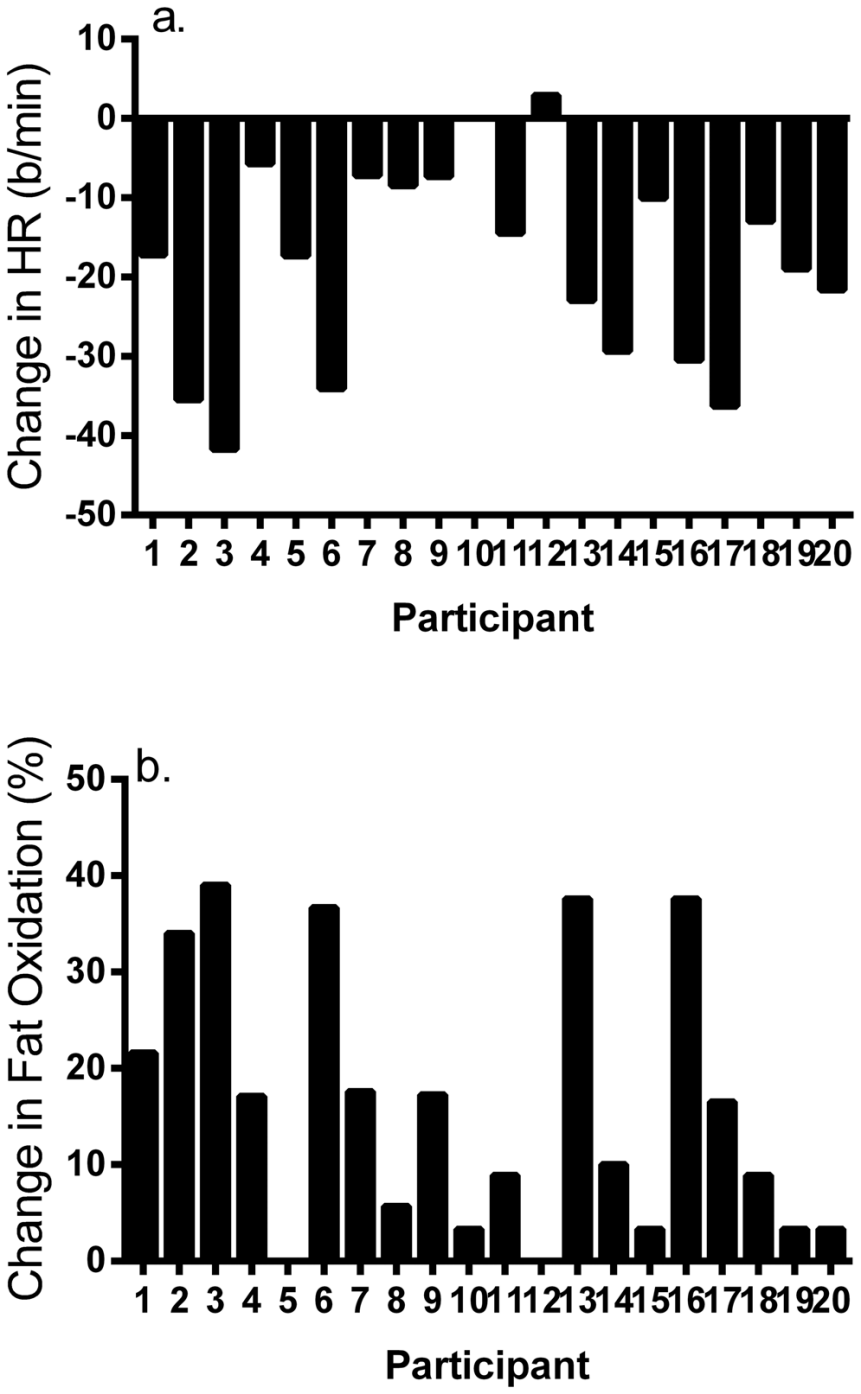
Individual responses for a) change in exercise HR and b) lipid oxidation in response to 12 wk of high-volume interval training.

Figure 4a demonstrates individual changes in exercise HR in response to high-volume interval training. Mean change in exercise HR was equal to −17±13 b/min (−28–−7 b/min, range = −42–3 b/min), with marked individual variability across participants. Seventeen of 20 participants (85%) showed reductions in exercise HR in response to training; whereas, 15% were classified as nonresponders.

#### Individual changes in fat oxidation

Individual changes in whole-body fat oxidation derived from RER are demonstrated in Figure 4b. Mean change in fat oxidation was equal to 16.8±14.4% (95%CI = 9.6–24.0%, range = 0.0–39.0%). Sixty five percent of women (13/20) revealed improved fat oxidation in response to 12 wk of HIT, and 35% showed minimal or no change in this parameter.

#### Multiple regression data

A model (R = 0.71, p = 0.003) consisting of baseline VO_2_max (r = −0.63, p = 0.002) and body fat explained 50% of the variance in the change in VO_2_max after 12 wk of training. A two-predictor model (R = 0.80, p = 0.001) consisting of baseline VO_2_max and HR at 40 W significantly explained 64% of the variance in the training-induced reduction in HR. Besides VO_2_max (r = 0.52, p = 0.008) and HR at 40 W (r = −0.75, p = 0.000), significant correlates of the change in exercise HR also included age (r = −0.44, p = 0.02), body fat (r = −0.44, p = 0.02) and HR at 60 (r = −0.69, p = 0.001) and 80 W (r = −0.57, p = 0.004). Many predictors were significantly related to increases in whole-body fat oxidation, including baseline VO_2_max (−0.44, p = 0.03), body fat (r = 0.40, p = 0.04), age (r = 0.45, p = 0.02), waist circumference (r = 0.38, p = 0.05), waist:hip ratio (r = 0.47, p = 0.02), and RER at 40 W (r = 0.51, p = 0.01). Waist:hip ratio and RER at 60 W (R = 0.66, p = 0.008) explained the greatest variance (43%) in improvements in fat oxidation.

#### Overall changes in VO_2_max, HR, and fat oxidation

Tables 1 and 2 show frequency of participants revealing significant changes in measured parameters in both studies. Six of 20 participants (30%) in study 1 revealed improvements in all parameters; similarly, six (30%) showed beneficial changes in two of three variables and seven (35%) revealed adaptation in only one parameter. One male participant (age = 23 yr, VO_2_max = 52.3 mL/kg/min) whose VO_2_max was highest in this sample was a “non-responder” for all measures. Eleven women (55%) in study 2 were classified as responders in all outcome measures; whereas, seven (35%) demonstrated adaptation in two variables and the remaining two women (10%) were classified as responders for only one measure (VO_2_max).

**Table 1:** Individual responses in VO_2_max, exercise HR, and fat oxidation to low-volume HIT in active men and women.

Participant	1	2	3	4	5	6	7	8	9	10	11	12	13	14	15	16	17	18	19	20	Response Frequency
**Parameter**																					
VO_2_max	R	R	NR	R	R	NR	R	R	NR	R	R	R	NR	R	NR	NR	R	R	R	NR	65%
eHR	NR	NR	R	NR	R	R	R	R	NR	R	R	NR	NR	NR	R	NR	R	NR	R	R	55%
Fat oxidation	NR	NR	R	NR	R	R	NR	R	R	R	R	R	R	R	NR	NR	R	NR	R	R	65%

R = responder; NR = non-responder; eHR = exercise heart rate; note that the columns refer to individual participants.

Low-volume HIT = 2 weeks (6 sessions) of Wingate-based HIT.

**Table 2:** Individual responses in VO_2_max, exercise HR, and fat oxidation to high-volume HIT in sedentary women.

Participants	1	2	3	4	5	6	7	8	9	10	11	12	13	14	15	16	17	18	19	20	Response Frequency
**Parameter**																					
VO_2_max	R	R	R	R	R	R	R	R	NR	R	R	R	R	R	R	R	R	R	R	R	95%
eHR	R	R	R	NR	R	R	R	R	R	NR	R	NR	R	R	R	R	R	R	R	R	85%
Fat oxidation	R	R	R	R	NR	R	R	NR	R	NR	R	NR	R	R	NR	R	R	R	NR	NR	65%

R = responder; NR = non-responder; eHR = exercise heart rate; note that the columns refer to individual participants.

High volume HIT = 12 weeks of 3 sessions/wk of 6–10 1-min bouts @ 60–90% W_max._

## Discussion

The primary aim of this retrospective study was to separately examine individual responses to two regimens of high-intensity interval training (SIT and HIT) performed by young, healthy men and women varying in fitness level. Changes in VO_2_max, heart rate, and fat oxidation were identified as they are frequently assessed in response to completion of endurance [5], [31]) and/or interval training interventions [14]–[15] and are related to cardiovascular and metabolic health. Results demonstrated that prolonged, high-volume HIT elicits greater frequency of adaptations in VO_2_max and reduction in HR and lower frequency of “non-responders” compared to prolonged endurance training [4], [5]. In contrast, two weeks of low-volume SIT demonstrated high frequency of non-responders (35–45%) in all variables. Predictors of change in these variables included age, baseline VO_2_max and fatigue index, and current physical activity as well as waist:hip ratio and measures of HR and RER obtained during moderate exercise. Overall, participants desiring to potentially improve VO_2_max through interval training may perform more prolonged, higher volume regimens of HIT as the magnitude of change in VO_2_max and frequency of nonresponse are lower than those frequently reported for endurance training. Nevertheless, frequency of non-responders to all outcomes was low (1 of 40 individuals), suggesting that either HIT or SIT provides a robust stimulus to improve cardiorespiratory fitness and metabolic health in young men and women.

Our data align with recent findings from two meta-analyses documenting effects of interval training on VO_2_max in young, active adults. Gist et al. [32] demonstrated a small to moderate effect of 2–10 wk (mean duration = 4.8±2.3 wk) of Wingate-based SIT on VO_2_max, as shown by 8% and 3.6 mL/kg/min improvements in VO_2_max compared to controls, although the effect was similar to that of endurance training and quite heterogeneous across studies. Our lower percent change in VO_2_max in response to Wingate-based SIT is likely due to its relatively brief duration, supporting data from Bailey et al. [33] revealing a 6.7% improvement in VO_2_max after only 2 wk of training. Bacon et al. [34] summarized 37 studies conducted in untrained men and women (VO_2_max <55 mL/kg/min) performing a minimum of 6 wk of HIT and 10 min of training per session. These authors reported larger increases in VO_2_max (0.51 L/min, 95% CI = 0.43–0.60 L/min) than previously reported via interval training [15], [17], [21] or from the current study (0.39 L/min, see Results). In addition, they cited that studies employing longer intervals (>3 min) combined with endurance exercise producing a greater training volume typically led to greater changes in VO_2_max than those characterized by shorter bouts and lower volume. This intuitively makes sense, as completion of longer duration exercise, albeit at intensities approaching or at VO_2_max, is more dependent on aerobic metabolism than ≤60 s bouts more reliant on nonoxidative metabolism. Overall, these authors concluded that higher volume (>10 min of exercise) interval training increases VO_2_max in most young individuals, with greater increases in VO_2_max seen compared to results from large-scale training studies [3]–[5] revealing a high frequency of non-responders to training. Our data support this conclusion, as we show that low-volume SIT tends to elicit lower frequency of increases in VO_2_max (65%) than via endurance training. The fact that low-volume SIT seems to induce mostly peripheral versus central cardiovascular adaptations [35] is a plausible explanation for this discrepancy as well as the habitually active status of the majority of individuals participating in these studies.

Results from both interval training regimens showed that baseline VO_2_max was inversely related to training-induced change in VO_2_max. In contrast, in the HERITAGE study [7], participants with high and low VO_2_max revealed similar increases (348–419 mL/min) in this parameter. In fact, age and gender were the best predictors, with baseline VO_2_max explaining only 1% of the variance in VO_2_max response to training. Yet in a different subset of data from this study reported by Skinner et al. [36], a significant inverse correlation (r = −0.38) occurred between baseline VO_2_max and percent change in this measure. Similar lack of significant associations between these baseline factors and change in VO_2_max was shown in middle-aged men and women performing 1 yr of endurance training [5] and men and women aged 60–71 yr performing 9–12 mo of endurance training [2]. Differences in participants’ age and baseline fitness level and mode, frequency, and duration of exercise across studies could explain these dissimilar results. For example, our participants were much younger than the individuals recruited in these aforementioned studies. In contrast, data from the DREW study [4] revealed that baseline VO_2_max was a significant predictor of VO_2_max response to 6 mo of continuous training in postmenopausal women. In addition to baseline VO_2_max, data from the current study showed that body fat was inversely correlated to change in VO_2_max in sedentary women completing 12 wk of HIT; whereas, Wingate-derived fatigue index ((peak power – minimum power)/peak power) was positively correlated with change in VO_2_max in active men and women performing low volume SIT. This may suggest that body composition and fatigue resistance influences resultant changes in VO_2_max to interval training, although further study is merited to confirm this assumption. Overall, it is plausible that no relationship between baseline VO_2_max and resultant change in VO_2_max is likely in homogeneous populations; whereas, significant relationships may be detected when participants are heterogeneous, as was the case in our studies.

Despite the robust increase in VO_2_max reported by Bacon et al. [34], their results are somewhat diminished by the extensive time commitment needed to optimize VO_2_max. One of the main advantages of low-volume SIT is its relative time-efficiency both in actual exercise time (∼ 2–10 min/session) as well as total session time typically less than 30 min. Recent data in sedentary women [25] show that increases in VO_2_max were comparable whether more (80–90%Wmax) or less intense (60–80%Wmax) regimens of HIT were performed for 12 wk, suggesting that a greater intensity of interval training following an identical regimen (mode, duration, number of bouts, frequency, etc.) may not maximize changes in VO_2_max, as was revealed versus moderate exercise in athletes [37]. Moreover, HIT elicits similar adaptations as continuous exercise [21] yet has been perceived as more enjoyable [24] which in the long run may promote exercise adherence and ultimately greater gains in fitness and health outcomes. Nevertheless, does a small additional increase in VO_2_max exhibited with more prolonged interval training justify the extra time allotment, based on the fact that lack of time [38] is often cited as the greatest barrier to exercise? Despite empirical data [39] showing that greater values of VO_2_max reduce future risk of chronic disease, the minimal time commitment and efficacy of short-term SIT (bouts ≤1 min) described in various populations may position it as a more desirable alternative to regimens such as endurance training requiring greater than 30 min/d.

Compared to VO_2_max and fat oxidation, there was lower frequency of adaptation in exercise HR in response to Wingate-based SIT. In the HERITAGE study [31], 20 wk of endurance training decreased HR during cycling at 50 W by 11 b/min, although individual responses ranged from reductions in HR as large as –42 b/min to a 12 b/min increase. Their data [7] also showed that participants with elevated pre-training HR at this workload demonstrated larger decreases in HR compared to those with a lower exercise HR (−16 b/min vs. −7 b/min, respectively). In addition, baseline HR at 50 W and gender were significant predictors of change in exercise HR, with ethnicity and age having minimal relationships. Our data support this evidence as in response to both HIT paradigms, submaximal HR obtained pre-training was a strong, significant predictor of its response to training. Recently, Rankinen et al. [6] showed that heritability of HR response to training was localized to nine single-nucleotide polymorphisms (SNPs) related to cardiomyocyte and neuronal function. Overall, practitioners should not expect marked reductions in HR in clients with an existing blunted response to exercise as typically seen in habitually active individuals.

Although predictors of change in VO_2_max and exercise HR in response to endurance training have been identified, less is known regarding correlates of changes in fat oxidation. In one cross-sectional study, Stisin et al. [12] compared fat oxidation between untrained (VO_2_max = 41.5 mL/kg/min) and trained women (VO_2_max = 53.8 mL/kg/min) during progressive exercise. Although maximal fat oxidation (in g/min) and workload coincident with maximal fat oxidation were similar between groups, trained women showed higher fat oxidation at moderate and high intensities versus untrained women. Compared to the untrained women, trained women revealed higher (p<0.05) activities of citrate synthase(CS), hormone sensitive lipase, and beta-hydroxy acyl CoA dehydrogenase (B-HAD). Across all women, significant positive relationships were exhibited between CS/B-HAD and the workload coincident with maximal fat oxidation as well as fat oxidation at 150 W. In a previous study identifying determinants of RER [13], trained cyclists exercised at 25, 50, and 70%Wmax which was accompanied by measurements of blood lactate, free fatty acids, as well as substrate concentrations via muscle biopsy. Results demonstrated that training volume, muscle glycogen, percent type I fibers, and lactate and free fatty acid concentration were key determinants of exercise RER, supporting Stisin et al.’s [12] data. Our results add to the literature by showing that noninvasive, widely-obtained measures including exercise RER, waist:hip ratio, and age and volume of physical activity can also be used to predict change in fat oxidation in response to interval training. Nevertheless, VO_2_max of individuals participating in our training studies was typically lower than that reported in previous studies, so generalization of our findings to more trained populations is cautioned.

One interesting finding of our study is that the frequency of improvements in whole-body fat oxidation was comparable between individuals in study 1 and 2 (see Results) despite the markedly different duration (6–9 min/wk vs. 18–30 min/wk) and intensity (200–300%Wmax vs. 60–90%Wmax) of training performed as well as discrepancies in VO_2_max, gender, and body composition across participants. In addition, the protocols used to assess fat oxidation across studies slightly differed. Low-volume SIT has been reported [22], [35] to induce primarily peripheral versus central cardiovascular adaptations that would increase fat oxidation, so it may be that mitochondrial signaling changes are more sensitive to the intensity of exercise rather than overall training volume. For example, mRNA content for primary regulators of mitochondrial biogenesis and lipid metabolism was similar in response to 90 min of moderate exercise compared to interval exercise at 120%VO_2_max [40]. However, large variability in exercise RER has been documented in trained athletes [10], [13], which suggests that training-induced changes in RER should vary across individuals. In addition, men and women in study 1 completed 30 min of cycling after a 3 h fast, yet in study 2, women underwent a 12 h fast before performing a shorter bout of progressive exercise, which may elicitt discrepancies in glycogen content and glucose/insulin levels across participants which affect substrate oxidation. All participants ingested widely divergent diets, so it is likely that circulating free fatty acid concentrations differed before exercise which may alter resultant substrate oxidation. In study 2, increases in fat oxidation peaked at 6 or 9 wk of training in many individuals and did not change or slightly declined at 12 wk, sofurther studies are needed to identify the optimal exercise regimen to sustain the improved fat oxidation observed with interval training.

There are a few implications of identifying individual responses to interval training. One, our findings corroborate data obtained from endurance-training studies [1], [4] that not every individual adapts to training despite steady increases in frequency, intensity, and/or duration of training. Second, it emphasizes that assessments should be done frequently after initiation of training, and in the case of interval training, after as little as 2 wk, to ensure that anticipated adaptations are occurring. If they are not, specific training parameters should be modified to promote potential for adaptation. To our knowledge, this unique approach has yet to be instituted with interval training and may optimize adaptation, even in individuals previously identified as “non-responders.” Lastly, it raises a compelling question: what can a practitioner do if, for example, VO_2_max does not increase with exercise training in an individual with known risk factors for chronic disease? Potentially other factors need to be targeted such as blood pressure, waist circumference, blood lipids, or even inflammation using a more individualized approach. We encourage scientists leading large-scale training studies to attempt to “follow-up” with non-responders to examine if other modalities of exercise training are effective to improve health status.

Limitations to this study include its use of dissimilar regimes of HIT, one employing Wingate-based SIT in active men and women and the other consisting of lower intensity bouts of high-volume HIT in sedentary women, although the regimens were analyzed separately and not compared. Clearly, additional data collection is merited in untrained and active individuals completing the same interval training regimen to compare the effectiveness of each protocol. In addition, all participants were young and free of disease, so it is likely that individual responses may differ in older adults, clinical populations, as well as persons who are extremely deconditioned. Exercise was performed on a cycle ergometer which was a relatively unfamiliar mode of exercise for most participants, so adaptations to treadmill training may have varied. No mechanistic variables were obtained in either study, such as glucose tolerance (insulin sensitivity and fasting insulin), hemodynamic function (stroke volume and cardiac output), or muscle oxidative capacity (fiber type expression, citrate synthase, etc.), whose changes may parallel individual changes in VO_2_max, HR, and fat oxidation. Fat oxidation is affected by habitual fat intake [13], and although dietary intake was standardized for 24 h before assessments, regular dietary practices were not considered in regards to altering the change in fat oxidation. Our use of the coefficient of variation of various measures to identify responders and non-responders has precedence [5], yet does not include random error in the measurement. Therefore, frequency of response to interval training may be slightly overestimated in the current study. However, this study is strengthened by inclusion of a large, heterogeneous sample of men and women differing in ethnicity, BMI, fitness level, and body fat as well as stringent control of workloads completed, continuous supervision of all exercise sessions, and high compliance rate.

## Conclusions

Results from this retrospective analysis of 40 individuals completing different interval training regimes indicated that the frequency of non-responders was 5–45% depending upon the parameter measured and specific regimen completed. In addition, our results suggest that a 12 wk regime of HIT elicits superior individual responses in VO_2_max and exercise HR compared to that reported from endurance training [5] as well as low-volume SIT [18], [21]. Compared to improvements in VO_2_max and exercise HR, frequency of improvement in exercise fat oxidation was typically lower, therefore further research is warranted to identify the optimal regimen of interval training to improve fat oxidation. In summary, for individuals who desire to improve their health but have low levels of cardiorespiratory fitness, beginning an exercise-based weight-maintenance or weight-loss program with HIT could increase probability of improving VO_2_max before transitioning into moderate-intensity endurance exercise with the goal of improving body composition and metabolic function.
